# Immunological Aspects of Dental Implant Rejection

**DOI:** 10.1155/2020/7279509

**Published:** 2020-12-09

**Authors:** Milad Baseri, Faraz Radmand, Reyhaneh Hamedi, Mehdi Yousefi, Hossein Samadi Kafil

**Affiliations:** ^1^Dental and Periodontal Research Center, Tabriz University of Medical Sciences, Tabriz, Iran; ^2^Biotechnology Research Center, Faculty of Dentistry, Tabriz University of Medical Sciences, Tabriz, Iran; ^3^Dental School, Tabriz University of Medical Sciences, Tabriz, Iran; ^4^Research Center for Pharmaceutical Nanotechnology, Tabriz University of Medical Sciences, Tabriz, Iran; ^5^Stem Cell Research Center, Tabriz University of Medical Sciences, Tabriz, Iran; ^6^Drug Applied Research Center, Faculty of Medicine, Tabriz University of Medical Sciences, Tabriz, Iran

## Abstract

Nowadays, dental implants are a prominent therapeutic approach among dentists for replacing missing teeth. Failure in dental implants is a severe challenge recently. The factors which lead to dental implant failure are known. These factors can be categorized into different groups. In this article, we discussed the immunological aspects of implant failure as one of these groups. Cytokines and immune cells have extensive and various functions in peri-implantitis. The equilibrium between pro and anti-inflammatory cytokines and cells, which involve in this orchestra, has a crucial role in implant prognosis. In conclusion, immune cells, especially macrophages and dendritic cells, almost increased in the patients with implant failure. Also, proinflammatory cytokines were proposed as diagnostic factors according to their higher levels in dental implant rejection.

## 1. Introduction

**Figure 1:**
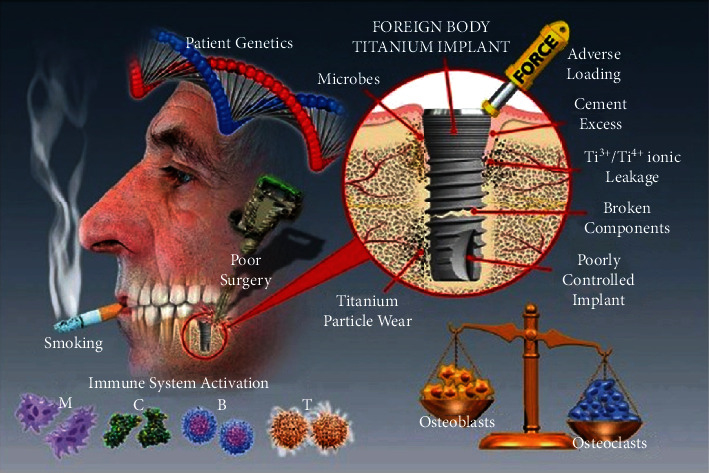
Factors associated with dental implant failure with cooperation of immune reactions. M: macrophages; C: complement activation; B: B-cells; T: T-cells. (Reproduced with permission from Albrektsson et al. [100]).

Dental implants are ordinarily used in clinics for the replacement of teeth. Even though many advances have occurred in materials, techniques, also in the design of the implant, implant failure in treatment is a primary concern for dentists and patients [1, 2]. “A graft that sets tightly or deeply into the alveolar bone.” is defined as an implant. Implants are utilized for single tooth replacements, partially or entirely edentulous arches [3]. Implant failure is narrated as an implant that shows a hopeless prognosis like a clinically mobile implant, implant displaying continued osseous support loss, fractured implants, and a bone loss, which extends at vital anatomic structures, further the implants that are not suitable enough to be utilized for restoration [4]. Many factors took part in implant failure such as peri-implantitis, lack of osseointegration, and also implant fracture. Moreover, it may happen because of surgical trauma, micromovement, and overloading, patient's medical history, smoking, and poor design of the implant, inappropriate selection of the patient, staff responsibility, poor oral hygiene due to accumulation of bacterial plaque, improper prosthetic restoration, accumulation of debris, and bone preparation without using any coolants [4–7] (Figure 1).

**Figure 2:**
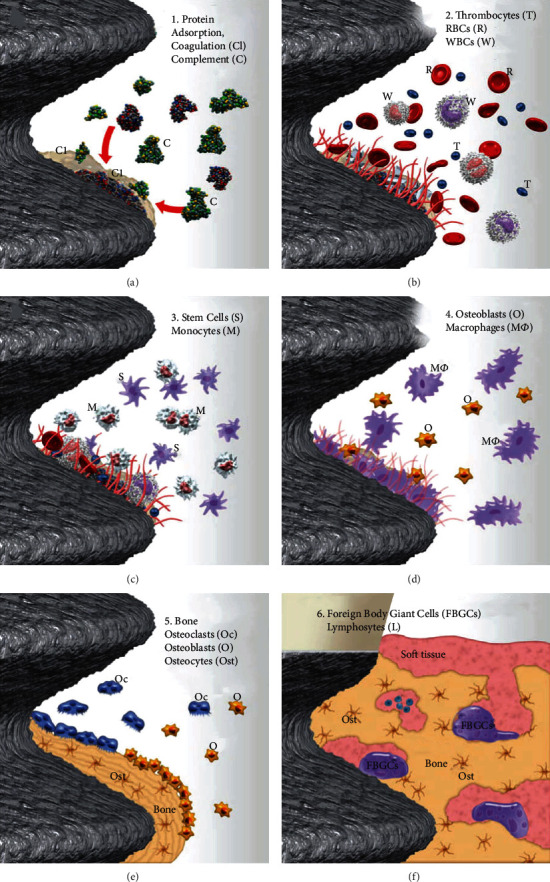
(a–f) Immune system and inflammation's role in osseointegration build-up RBC: red blood cell; WBC: white blood cell (Reproduced with permission from Albrektsson et al. [9]).

The longevity of dental implants depends on integration between the implant and both hard and soft tissues [4]. Osseointegration is an underlying issue for success in dental implants as replacements for patient's teeth. Osseointegration is “a procedure in which alloplastic materials' rigid fixation is obtained and maintained in the alveolar bone during functional loading” [8]. Osseointegration is influenced by the osteogenesis in the implant interface. This dynamic procedure is the result of intricate inflammation-relevant reactions, like bone apposition and resorption, neurogenesis, and angiogenesis [9] (Figure 2). The immune system reaction and inflammation require well-engaged and active biochemical processes to restore homeostasis, which consequently leads to osseointegration of implant [10]. The immune response has some significant factors such as cytokines, soluble substances produced by different kinds of immunocompetent cells whereby the cells affect each other [11]. Up to this date, there are several studies carried out in the field of implant rejection from the immunologic viewpoint and the alterations in the peri-implant environment.

The present study is aimed at evaluating the immunological factors of dental implant failure in a new and comprehensive way. In this study, the immunological reactions have been investigated from three aspects: cellar, humoral, and allergy. This classification of the subjects in this paper rises from the fundamental differences in the mechanisms of each part. Cellular and humoral immunity are principals of the immune system, and allergy is a subject which these two principles overlap on each other, so they are classified separately. Due to our knowledge, there was no in-depth research on this subject as we do. Our desire is to present an executive protocol for dentists all over the world to enhance the success rate of implant-based treatments.

## 2. Cellular Immunity

Poor osseointegration and chronic inflammation are two primary factors, which cause dental peri-implantitis [12]. Infiltration of immune cells as an important part of immune responses significantly affects the biocompatibility and function of dental implants and can lead to failure [13, 14]. The initial injury of peri-implant tissue triggers an inflammatory response mediated by the cells of innate immunity, such as macrophages, dendritic cells (DC), mast cells, and neutrophils.

**Figure 3:**
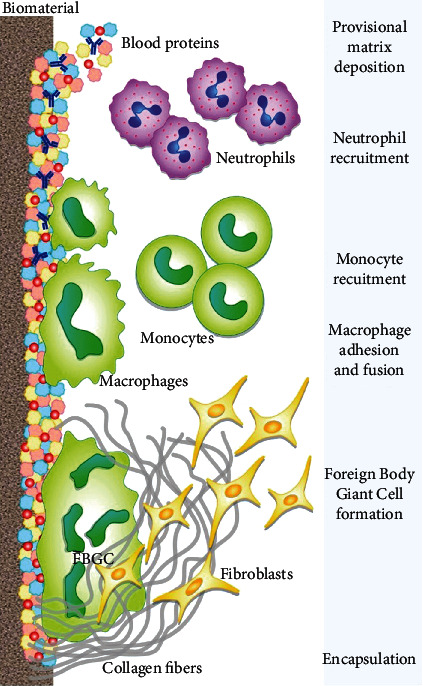
Macrophages are the principal cells in the innate immune reactions to biomaterials. Also, other cells like fibroblasts interact with macrophages in this process. (Reprinted with permission from Mariani et al. and International journal of molecular sciences [101]).

**Figure 4:**
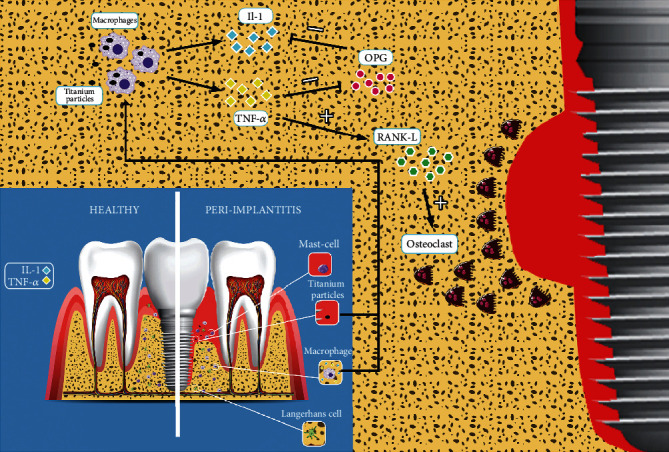
The interaction between immune cells and cytokines in healthy and peri-implantitis site.

Macrophages are the principal cells in the innate immune reactions to implants. When the body exposes to the implant material, the primary phagocytes which are activated in the early inflammation stage are the macrophages [15] (Figure 3). They have an indispensible role in the osseointegration of implants to the host recipient and delineate the fate of the implant [16, 17]. Macrophages release some cytokines, such as interleukin 1 (IL-1) and tumor necrosis factor-alpha (TNF-ɑ) activating the osteolytic and inflammatory process of peri-implantitis [18] (Figure 4). When foreign bodies such as dental implants are inserted in tissues, macrophages play a dual role by either inflammatory (M1 macrophages) or anti-inflammatory (M2 macrophages) responses [19]. Fretwurst et al. concluded that M1 macrophages were dominant cells in patients with peri-implantitis [20]. Furthermore, Wang et al. showed that peri-implant bone loss in a murine model had a positive correlation with M1 macrophage presence [21]. In another study, the authors found out the association between the presences of macrophages in rejected implants with the granulation tissue formation. They found metallic particles of titanium in macrophages' cytoplasm in the site of granulation tissue [22]. Also, multiple studies revealed that the presence of M2 macrophages in peri-implant tissue is related to reduction of inflammation, improvement in wound healing, and finally successful osseointegration of implants [23–25]. Chehroudi et al. suggested that enhancement of implants' osseointegration was related to macrophages accumulation showing their effect on new bone formation [26]. In conclusion, macrophages might have a binary role in directing the implant to failure or success. Once M2 macrophages could lead to osseointegration and effective wound healing, on the other hand, the M1 macrophages could exacerbate the inflammation process and accelerate osseolysis leading to dental implant failure.

DCs are main antigen-presenting cells in the activation of native T cells and coordination of the immune response, which are abundant at the osteoimmuneinterface [27]. Gooty et al. indicated that the number of DCs (factor XIII) in the epithelium and lamina propria of healthy mucosa which is obtained before the implant placement was significantly lower than healthy peri-implant mucosa which is obtained at the time of placement of the gingival former. Authors found that titanium particles seen around peri-implant tissues are due to corrosion of dental implants. Also, collagen degradation happens according to a higher number of factor XIIIa DC [28]. These results were the comparison between the healthy peri-implant tissues before and after insertion of the implant. Further studies are needed to achieve a remarkable consequence about the exact effect of these cells on dental implant rejection.

Langerhans cells (LCs), a type of dendritic cells, could be found in stratified epithelium such as the skin epidermis and the oral mucosa epithelium [29]. They can adjust the immunological environment of the oral mucosa and also preserve oral tissues during infection [30]. Various studies demonstrated the reduction or no significant alteration of LCs in the peri-implant tissues [31–33]. Hovav and Wilensky et al. demonstrated that whereas the percentage of LC precursors (CD11c + MHCII+) increased in the peri-implant epithelium, the frequencies of LCs (CD11c + MHCII+EpCAM+langerin+) were significantly reduced. They suggested that implants could impair the maturation of LCs and dysregulate immune responses in the peri-implant tissue [34]. Gooty et al. also indicated that the number of LCs (factor CD1a) in the epithelium and lamina propria of healthy mucosa which is obtained before the implant placement was significantly higher than healthy peri-implant mucosa which is obtained at the time of placement of the gingival former. The investigators deduced that decreased immune reactions are consequences of a lower number of LCs CD1a in the of peri-implant tissue [28]. Collectively, due to studies, the number of mature LCs is reduced due to dental implant insertion. However, the implants have a reverse effect on precursors LCs and could increase their number in peri-implant tissue.

Neutrophils are predominant and initial cells in peri-implant tissues. They participate in the inflammation process by various factors including cytokine and chemokine production and DNA fiber networks called neutrophil extracellular traps (NETs) [35]. Broggini et al.'s study showed that neutrophils are maximally aggregated coronal to the osseoimplant interface [36]. However, the role of neutrophils is undeniable in inflammation, and there are few studies done about their role in dental implant rejection. There is just a study investigating the relation between these cells and implant surface. Neutrophils release fewer proinflammatory cytokines, and NETosis does not occur when exposed to the rough-hydrophilic surface of the implant. The reduction of the proinflammatory accumulation of macrophages in response to NETs eventuates to successful osseointegration [35].

Mast cells (MCs) are granular cells derived from bone marrow hematopoietic cells, circulating in blood vessels, and releasing in tissues. Thus, they are involved in inflammation, tissue repair, and host defense. MCs play a protective role by activating defense mechanisms, osseointegration, and initiating tissue repair if their activity is transient, but if it is inappropriate and continuous, leading to osseointegration absence or peri-implantitis, it will lead to remarkable tissue damage [37]. Mast cells release interleukin-4 and 13 which determines the expanse and severity of the following development of the foreign body reaction [38, 39]. The effect of mast cells on implant failure is unclear due to the lack of sufficient studies in this field.

As a conclusion, innate immune cells had a significant role in reaction to dental implants, but on the other hand, we face a lack of considerable investigations about the role of adoptive immune cells in dental implant rejection. In order to increase the longevity of dental implants, monitoring the number of innate immune cells may be useful in preventing rejection. Also, more researches should be done to identify the exact role of adoptive immune cells in dental implant rejection.

## 3. Humoral

Most of the interrelation between cells of the immune system is modulated by several factors such as cytokines, growth factors, and hormones. Cytokines have an undeniable role in the intercommunication of the immune cells in the inflammatory process, which eventuate clinical manifestations [40]. Cytokines are messengers in cell to cell interaction that are vital for the pathogenesis of several diseases involving peri-implantitis [41]. They can be generally classified as proinflammatory and anti-inflammatory.

### 3.1. Proinflammatory

Interleukin-1 is a proinflammatory cytokine, which is produced by macrophages, neutrophilic granulocytes, and other cells [42]. IL-1*α* and IL-1*β*, which are isoforms of interleukin-1, have been strongly related to type 1 collagen's downregulation in bone and osteoclast activation. Therefore, cooperating in the normal bone resorption of peri-implantitis [40], IL-1*α* is expressed in many types of cells in healthy tissues permanently, but its expression can be raised in response to proinflammatory, growth factors, and stress-associated stimuli [43]. IL-1*β* is a kind of proinflammatory cytokine that contributed to numerous biologic processes, such as immune regulation, connective tissue metabolism, inflammation, the production and maturation of osteoclasts, and activation of mature osteoclasts to inhibit bone formation by resorbing bone [44, 45]. The level of IL-1*β* around peri-implantitis lesion has a positive influence on the quantity of gingival inflammation, demonstrating that it is a reliable indicator to diagnose peri-implant mucositis in an early stage before it advances to peri-implantitis [46, 47]. In this unique microenvironment, IL-1*β* starts the inflammation process and can play a protective and signaling role in the appearance of bacterial biofilms mostly by having a direct effect on the immune reaction to the infections [48]. IL-1ß also has control over the production of prostaglandin E2, which may play an essential role in the stimulation of hard tissue breakdown [49].

Further evidence proves that there was a positive correlation between the levels of IL-1 and the failure of dental implants, showing a complete profile of host reaction in patients with peri-implant collapse [50]. Moreover, IL-1*β* is known as a decisive factor for differentiating healthy implants and peri-implantitis from each other [42, 51]. Plenty of studies showed that the level of IL-1*β* in PICF is increased in patients with peri-implantitis condition [18, 41, 49, 52–56]. Despite this, Podzimek et al. found that the levels of IL-1*β* were considerably increased in patients with healthy implants in comparison to patients with failed implants after stimulating by titanium and mercury. Their notion toward the increase of Il-1*β* differs from the other studies due to the necessity of upregulation for the healing process. Subsequently, they hypothesized that decreased amounts of these cytokines may result in an implant failure in patients who have failed titanium implants [11].

TNF-*α* initiates the inflammatory cascade's essential mediators in correlation with IL-1*β* [57]. The TNF-*α* stimulates prostaglandin synthesis, bone resorption, and protease production through many cells such as osteoblasts and fibroblasts. The early release and rapid synthesis of TNF-*α* guide other cells to the infection site and microbial invasion [58]. High amounts of TNF-*α* have been found in regions with peri-implantitis and periodontitis. These findings proved that extreme secretion of TNF-*α* is an important clinical issue for acute or chronic inflammatory diseases [10]. Two mechanisms are induced by TNF-*α*: (1) directly by enhancing osteoclast precursors in the bone marrow and (2) indirectly by impression on osteoprotegerin (OPG)/RANKL system [59] (Figure 1). Remarkably increased levels of TNF-*α* were observed in patients with failed implants in comparison to patients with healthy implants. This enhancement of TNF-*α* causes dental implant failure [10, 11, 18, 41, 42, 51, 53, 55, 59–62]. Thus, TNF-*α* and IL-1*β* have been proposed as relevant biochemical markers due to their increased concentration in Peri-Implant Crevicular Fluid (PICF) of affected sites [40, 42].

IL-17, which immune reaction is mediated by Th17 cells, is another kind of proinflammatory molecule that takes part in excessive inflammatory actions [63] such as macrophage and neutrophil recruitment and some other proinflammatory mechanisms' stimulation [42]. Levels of IL-17 are high in patients with peri-implantitis, which proved that this cytokine augments local inflammation [42, 59, 60, 64].

Remarkably, IL-6 and IL-17can play both protective and destructive roles in tissues [65]. IL-6 is a cytokine that stimulates immune reactions. The main functions of IL-6 are acute-phase reactions and hematopoiesis, such as B cell growth, activation of T cell, and platelet production. Deregulation of IL-6 secretion has been associated with the pathogenesis of distinct kinds of chronic inflammatory and proliferative diseases [10, 66]. IL-6 participates in the controlling of differentiation, proliferation, and migration of cells. It is vital for the fixation of prostheses [67]. Moreover, the vast quantity of IL-6 production and also inflammatory response is correlated to the great tissue trauma [10]. Levels of IL-6 are high in failed implants in comparison to healthy implants [11, 51, 53, 55, 60, 68–71]. However, Severino et al. concluded that there was no significant difference when comparing the levels of IL-6 between healthy and peri-implantitis conditions [64].

Interleukin-8 (IL-8) is another proinflammatory cytokine that can be secreted by endothelial cells in the osseoimplant surface [72]. IL-8 is a well-known chemokine, which has several functions in the immune system including selective recruitment and activation of neutrophils in the gingival sulcus [41]. It was observed that healthy patients have significantly lower amounts of IL-8 in PICF compared with groups with mucositis [41, 50]. However, Severino et al. concluded that there was no significant difference when comparing the levels of IL-8 between healthy and peri-implantitis conditions [64].

IL-2 is a cytokine that has a crucial role in promoting cell proliferation. Its increase not only exhibits improved fixation and roughness because of the acid treatment but also is helpful in the biological acceptance of the implant [60]. Moreover, one study showed that the upregulation of IL-2 might cause failure in implants [60]. Mannose-binding lectin level is another factor, which can contribute in inflammation [73] but no study investigated its role, and further studies are needed.

Collectively, the result of different studies in the field of cytokines and dental implants shows that titanium particles stimulate the expression and secretion of proinflammatory cytokines such as IL-1, TNF-*α*, IL-17, IL-6, IL-8, and IL-2. These upregulations might be related to the loss of osseointegration and implant failure. However, there are some studies that reported controversial results about the rise of these cytokines. Further investigations are needed to identify the real role of these substances in dental implant rejection.

### 3.2. Anti-Inflammatory

IL-10, a multifunctional cytokine, has various impacts on hematopoietic cells. The primary function of this cytokine is the elimination and limitation of inflammatory reactions. Therefore, IL-10 is a critical immune modulator [74] and plays a positive role in vivo regulation, dampens acute inflammatory feedbacks, and produces a negative feedback loop that decreases the inflammatory mediators' release [60]. It suppresses macrophage and Th17 responses by inhibiting the production of proinflammatory cytokines like IL-6 and TNF-*α* [75]. IL-10 is one of the primary endogenous suppressors of infection and bone resorption through suppressing osteoclastic differentiation [10]. Concentrations of IL-10, which is an anti-inflammatory cytokine, decrease in peri-implantitis [42, 49, 76]. But some studies reported that IL-10 increases in peri-implantitis [53, 62, 77]. However, Severino et al. concluded that there was no significant difference when comparing the levels of IL-10 between healthy and peri-implantitis conditions [64].

TGF-ß1 is an anti-inflammatory factor that can regulate inflammatory response and immunosuppression [78, 79]. Also, it takes part in local inflammatory reactions and wound healing [15]. Downregulation of TGF-*β* is seen in peri-implantitis in relation to Th-17 and IL-23 [42]. Though, Cornelini et al. stated that TGF-*β* expression is upregulated in epithelial layers and vessels of patients with failed implant comparing to the healthy patients, and the difference between groups was statistically significant [80].

As we reviewed, it is hard to designate the exact role of anti-inflammatory cytokines studied in the rejection of dental implants, especially TGF-ß1 and IL-10. Therefore, maybe investigators should pay more attention to this interesting field.

### 3.3. Other Factors

Metalloproteinases are collagenases that have a physiological function named “creating space” for cells directed to the insult site. On the other hand, when inflammation does not clear up, these molecules will destroy the tissues of peri-implant [40]. Metalloproteinases are including MMP-1, MMP-7, and MMP-8. MMPs, especially MMP-8, are the main metalloproteinase in periodontitis. Additionally, it takes part in inflammatory procedure by destructing basement membrane components and extracellular matrix. MMP-8 and collagenase-2 were known as early signs of peri-implant collapse [81]. Accordingly, they are related to the development of experimental modifications in the individual template of the host reaction in the peri-implant crevicular fluid [42]. Several kinds of MMP, like MMP2, MMP7, and MMP9, were severely upregulated in peri-implantitis samples. MMPs take part in degrading and remodeling Extra Cellular Matrix (ECM) molecules by splitting components of cell-matrix contacts and cell to cell junctions [82].

OPG is secreted from dental mesenchymal cells and takes part in a paracrine aspect as a decoy receptor binding to receptor activator of nuclear factor- (NF-) KB ligand (RANKL) on osteoblasts/stromal cells. Moreover, it may cause improvement in osseointegration by inhibiting IL-l ɑ [37]. RANKL is a key factor in osteoclast formation and activation [83, 84]. Prognostic factors in peri-implantitis are including Sclerostin, OPG, and RANKL [42]. Additionally, it was proved that significantly increased soluble RANKL and lower OPG concentrations were seen in peri-implantitis compared to the healthy control sites [62, 85] (Figure 4).

OPN is a kind of osteoimmunoinflammatory marker which is related to both mineralization and bone development further in infective inflammation, acting as an immune modulator through regulating cytokine production [86, 87]. Osteopontin (OPN) may play a decisive role in the production of IL-1*β* and apoptosis in peri-implantitis, as confirmed by the analysis of the patient's PICF and cell-culture examination, decreasing inflammation through proinflammatory cytokines' downregulation in peri-implantitis [88].

Analysis of cytokines in the peri-implant crevicular fluid (PICF) may be a good indicator to estimate the prognosis of dental implant treatment. Several studies confirmed the negative role of proinflammatory cytokines on the longevity of dental implants; therefore, checking these cytokine levels, especially IL-1*β*, TNF-ɑ, and IL-6, seems more reliable to predicting the fate of implants.

## 4. Titanium Allergy

Allergy is an acute immunological response that occurs during contact with a known antigen. Implant-related allergic responses are commonly affiliated with immediate-type I or most regularly with type IV delayed-type hypersensitivity; in an ionic form of them, metals can bond to native protein to form haptic antigens. Moreover, they can trigger the mastocytes' and basophils' degranulation that may result in fail implants [89–91]. The titanium also has an extensive utilization in medicine and dentistry, with a high percentage of success due to its vast resistance against corrosion, low allergic potential, low toxicity, and the desirable biocompatibility which is given by its passive stable oxide film. Additionally, titanium is the choice material in the medical field for intraosseous use [92].

**Figure 5:**
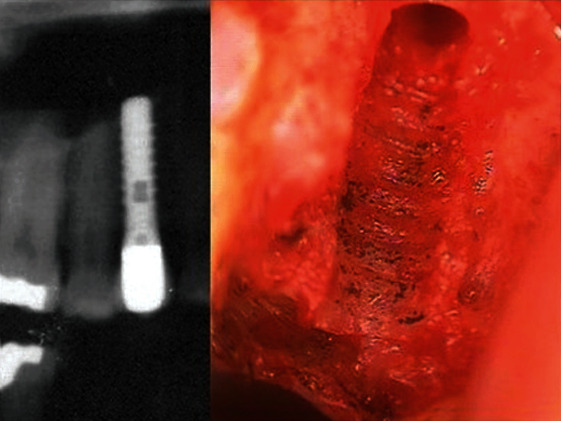
X-ray image of a failed implant (left panel). Titanium corrosion in the alveolar bone at the site of failed dental implant (right panel). (Reproduced with permission from Lechner et al. [102]).

The existence of metallic fragments in peri-implant tissues is because of simultaneous occurrence wear or corrosion and frictional wear which is called tribocorrosion [93]. Corrosion is the decomposition of metal that occurs gradually because of interaction with the ambient environment that gives rise to the release of some ions into the ambient tissues [94]. Corrosion has some effects such as substance loss from the material, structural integrity loss, changes in its structural characteristics, peri-implant soft tissues' discoloration, or type IV hypersensitivity responses, where the microparticles of titanium are observed inside macrophages [22, 95] (Figure 5).

Local existence of T lymphocytes and copious macrophages showing Type 4 hypersensitivity leads to the characterization of sensitivity to titanium [96]. Titanium causes the activation of macrophages directly or after phagocytosis, and activated macrophages secrete both anti and proinflammatory cytokines [92]. Bressan et al. proved that in vitro, titanium particles could influence mitochondria and induce ROS production. Also, they observed titanium particles in all peri-implant tissues [82]. There are various symptoms that range from skin rashes and implant failure to nonspecific immune suppression. However, we know that allergy to titanium is infrequent and that not all patients show sensitivity to metal after an endosseous implant [97].

**Figure 6:**
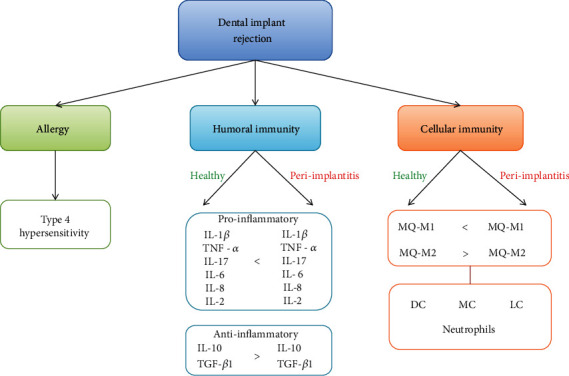
Summary of the immune system-related dental implant failure from three aspects of cellular and humoral immunity and allergic reactions.

There are various types of diagnostic tests to discover the titanium allergy. Type-1 allergy is diagnosed by some tests such as epicutaneous tests (patch tests), skin test (prick test), and the lymphocyte transformation test (LTT). The developed version of LTT is named Memory Lymphocyte Immuno Stimulation Assay (MELISA) [98, 99]. According to the evidence discussed above, nanoparticles of implant material are frequently observed in peri-implant sites. To prevent adverse effects of this nanoparticle accumulation, which leads to implant failure, using diagnostic tests will help to have a better prognosis. The results of this paper are summarized in the tree diagram (Figure 6).

## 5. Conclusion

Dental implant failure is a primary concern for dentists, which is avoidable by using evidence in the studies. One of the principle factors prompting implant failures is immunologically mediated rejection. This review demonstrated that immune cells, especially macrophages and dendritic cells, almost increased in the patients with implant failure. Also, proinflammatory cytokines were proposed as diagnostic factors according to their higher levels in dental implant rejection. Finally, the patient's titanium allergy should be evaluated in order to increase the success rate of treatment. Limitations were the lack of studies about adoptive cells and anti-inflammatory cytokines.
